# Supplementary material for: Deep learning with robustness to missing data: A novel approach to the detection of COVID-19
Source: PLoS One. 2021 Jul 30;16(7):e0255301. doi: 10.1371/journal.pone.0255301 (PMC8323880; doi:10.1371/journal.pone.0255301)
Supplement: S1 File — (PDF) [file pone.0255301.s001.pdf]

S1 Protocol. Data Cleanup In a small number of cases laboratory parameters are not specified with absolute numeric values, but rather by denoting that the value fell above or below a specific threshold (based on guidelines from each institution). To obtain numeric values we converted these using the protocol shown in Supporting Information S1 Table. Any values of Potassium, Lactate Dehydrogenase, and Magnesium that were considered as an outlier by the institutions were removed.

S1 Table. Data Cleanup Data cleanup protocol thresholds and replacement values **are provided in Table 4.**

Table 4. Data cleanup protocol thresholds and replacement values.

Parameter	Specified as	Converted to
C-reactive protein	< 3 mg/L	2 mg/L
D-Dimer	> 4 mg/L	4.01 mg/L
D-Dimer	< 0.17 mg/L	0.16 mg/L
Gamma-Glutamyl Transferase	< 5 U/L	5 U/L
Procalcitonin	< 0.03 ng/ml	0.01 ng/ml
Procalcitonin	> 75 ng/ml	76 ng/ml

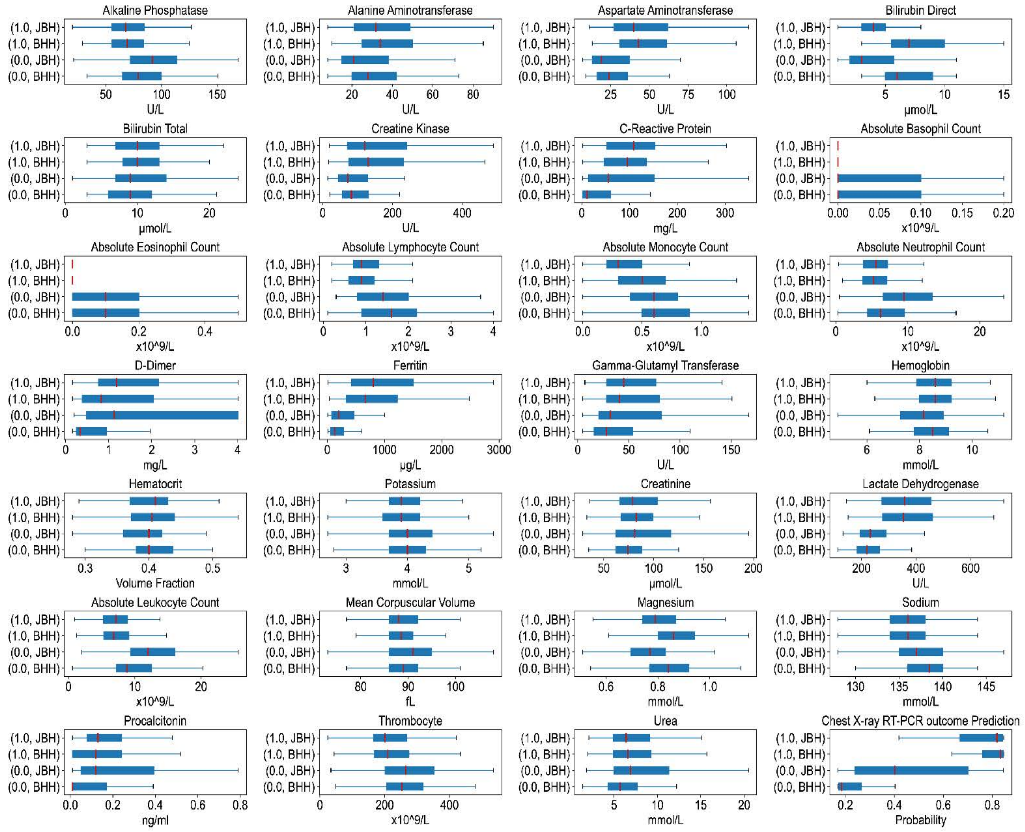

Value ranges of the 27 laboratory test results and chest x-ray scores, separated by institute and by positive/negative RT-PCR test results. JBH stands for Jeroen Bosch hospital and this data is used as the test dataset, BHH stands for Bernhoven Hospital and the data from this institution is used as the training dataset in our study. Positive cases are indicated by 1.0 and Negative by 0.0. It should be noted that the chest x-xray scoring model was trained using data from BHH leading to a difference in the scores produced between instiututes.

S1 Fig. Data Distribution The input data distribution per hospital and per RT-PCR test outcome is provided.

S1 Text. Training Settings

RT-PCR test prediction from CXRs

Training settings

We train Resnet-18 using a cyclic learning rate [1] between 0.001 and 0.01 and 2.5 epoch step size. We use stochastic gradient descent with Nesterov momentum of 0.95. We minimize the cross-entropy loss between the softmax activations and binary labels. We perform all experiments with a batch size of 16.

When fine-tuning the last layer on RT-PCR test results, we use a heavy label smoothing regularization [2] of 0.2 to prevent overfitting. We apply a weight of 0.625 to the positive samples, to account for the class imbalance. This number is calculated by dividing the number of negative cases in the training dataset by the number of positive cases. After training, we restore the model weights that have achieved the best validation cross-entropy loss.

Image preprocessing

We convert the DICOM files to 8-bit PNG files by clipping the values above the 99th percentile and scaling the resulting values between 0 and 255. We resize the resulting images to 512 by 512. Before we feed those images to the convolutional neural network, we apply data augmentation to prevent overfitting. We augment the images by cropping a width and height in the range of (409, 512] randomly. We resize the resulting image to 448 by 448. We randomly scale (by $[0.75, 1.25]$) and shift (± 64) the pixel values and clip the values above 255. We randomly flip the image from left to right. We standardize the resulting pixel values using the ImageNet dataset means and variances.

Model Selection: Validation dataset experiments

Training settings

Similar to the previous experiment, we train DAE, SDAE, FCN, and DFCN using a cyclic learning rate [1] between 0.001 and 0.01 and 2.5 epoch step size. We use stochastic gradient descent with Nesterov momentum of 0.95. We minimize the cross-entropy loss between the softmax activations and binary labels. For all models we use ReLU activations for intermediate layers. We train our models until there is no improvement in the validation loss for 10 epochs. We perform all experiments with a batch size of 16. When reconstruction regularization is used, the loss is calculated using the sum of squared errors of the prediction differences, applied with a coefficient of 0.03. We normalize each of the 27 laboratory parameters to unit mean and variance with respect to the training dataset statistics. After normalization, we set the masked and missing values to zero. We calculate the reconstruction loss only using the known values that are not missing in the training dataset. We apply a weight of 0.625 to the positive samples to account for the class imbalance as described in Supporting information Training settings.

Random Forest Settings

We use the Random Forest implementation of Scikit-learn (v0.22.1) [3]. This implementation does not allow partial training or mini batching. Hence, we apply input masking by repeating the dataset 100 times and applying the input masking on this set. For comparability with the other methods, we impute the missing values using the mean. We use the default model parameters because they have performed the best in our preliminary experiments.

S2 Table. Robustness validation experiments expanded results The results of Table 2 is truncated for simplicity. Here in Table 5, we provide the full robustness validation experiment results.

Table 5. All robustness evaluation AUC results of experiment Model Selection: Validation dataset experiments.

	AUCs on heavily masked validation data				
**IMP**	**DAE**	**SDAE**	**FCN**	**DFCN**	**RF**
**NIM → 0.0**	0.811	0.746	0.799	0.763	0.796
**0.1**	0.798	0.806	0.810	0.812	0.816
**0.2**	**0.815**	0.810	0.817	0.822	0.785
**0.3**	0.810	0.818	0.822	0.823	0.792
**0.4**	0.783	**0.834**	0.834	0.832	0.799
**0.5**	0.795	0.834	0.838	0.836	0.812
**0.6**	0.800	0.828	**0.838**	**0.843**	0.830
**0.7**	0.789	0.827	0.834	0.827	**0.831**
**0.8**	0.783	0.730	0.833	0.808	0.816
**0.9**	0.681	0.497	0.822	0.809	0.797

S3 Table. All p values for significance tests in results Clinically relevant subsets A and B The statistical significances mentioned in results Clinically relevant subsets A and B are provided in Tables 6, 7, and 8.

Table 6. p values from comparing AUC results (DeLong's test) of experiment Clinically relevant subsets A and B on All test data. Models with statistical significance and significant p-values are presented in bold.

Model 1	Model 1 Training	Model 2	Model 2 Training	Test Dataset	p-value
DAE	All	**DFCN**	All	All	**0.00681**
DAE	All	FCN	All	All	0.58261
**DAE**	All	RF	All	All	**0.02875**
DAE	All	SDAE	All	All	0.16335
**DFCN**	All	DAE	All	All	**0.00681**
**DFCN**	All	FCN	All	All	**0.03849**
**DFCN**	All	RF	All	All	$<$ **0.00000**
DFCN	All	SDAE	All	All	0.23376
FCN	All	DAE	All	All	0.58261
FCN	All	**DFCN**	All	All	**0.03849**
**FCN**	All	RF	All	All	**0.00134**
FCN	All	SDAE	All	All	0.40531
RF	All	**DAE**	All	All	**0.02875**
RF	All	**DFCN**	All	All	$<$ **0.00000**
RF	All	**FCN**	All	All	**0.00134**
RF	All	**SDAE**	All	All	**0.00024**
SDAE	All	DAE	All	All	0.16335
SDAE	All	DFCN	All	All	0.23376
SDAE	All	FCN	All	All	0.40531
**SDAE**	All	RF	All	All	**0.00024**

Table 7. p values from comparing AUC results (DeLong's test) of experiment Clinically relevant subsets A and B on **Subset A** labels of the test data. Models with statistical significance and significant p-values are presented in bold.

Model 1	Model 1 Training	Model 2	Model 2 Training	Test Dataset	p-value
DAE	All	DAE	Subset A	Subset A	0.58384
DAE	All	**DFCN**	All	Subset A	**0.02982**
DAE	All	**DFCN**	Subset A	Subset A	**0.04600**
DAE	All	FCN	All	Subset A	0.13971
DAE	All	**FCN**	Subset A	Subset A	**0.02978**
DAE	All	RF	All	Subset A	0.56853
DAE	All	RF	Subset A	Subset A	0.90902
DAE	All	SDAE	All	Subset A	0.35907
DAE	All	**SDAE**	Subset A	Subset A	**0.02188**
DAE	Subset A	DAE	All	Subset A	0.58384
DAE	Subset A	DFCN	All	Subset A	0.10716
DAE	Subset A	DFCN	Subset A	Subset A	0.23022
DAE	Subset A	FCN	All	Subset A	0.32074
DAE	Subset A	FCN	Subset A	Subset A	0.11969
DAE	Subset A	RF	All	Subset A	0.35180
DAE	Subset A	RF	Subset A	Subset A	0.79816
DAE	Subset A	SDAE	All	Subset A	0.71616
DAE	Subset A	SDAE	Subset A	Subset A	0.17905
**DFCN**	All	DAE	All	Subset A	**0.02982**
DFCN	All	DAE	Subset A	Subset A	0.10716
DFCN	All	DFCN	Subset A	Subset A	0.58065
DFCN	All	FCN	All	Subset A	0.25026
DFCN	All	FCN	Subset A	Subset A	0.97176
**DFCN**	All	RF	All	Subset A	**0.00318**
**DFCN**	All	RF	Subset A	Subset A	**0.01887**
**DFCN**	All	SDAE	All	Subset A	**0.02356**
DFCN	All	SDAE	Subset A	Subset A	0.59272
**DFCN**	Subset A	DAE	All	Subset A	**0.04600**
DFCN	Subset A	DAE	Subset A	Subset A	0.23022
DFCN	Subset A	DFCN	All	Subset A	0.58065
DFCN	Subset A	FCN	All	Subset A	0.99266
DFCN	Subset A	FCN	Subset A	Subset A	0.54821
DFCN	Subset A	RF	All	Subset A	0.08873
DFCN	Subset A	RF	Subset A	Subset A	0.27054
DFCN	Subset A	SDAE	All	Subset A	0.50519
DFCN	Subset A	SDAE	Subset A	Subset A	0.78887
FCN	All	DAE	All	Subset A	0.13971
FCN	All	DAE	Subset A	Subset A	0.32074
FCN	All	DFCN	All	Subset A	0.25026
FCN	All	DFCN	Subset A	Subset A	0.99266
FCN	All	FCN	Subset A	Subset A	0.33117
**FCN**	All	RF	All	Subset A	**0.02002**
FCN	All	RF	Subset A	Subset A	0.09254
FCN	All	SDAE	All	Subset A	0.11815
FCN	All	SDAE	Subset A	Subset A	0.78720
**FCN**	Subset A	DAE	All	Subset A	**0.02978**
FCN	Subset A	DAE	Subset A	Subset A	0.11969
FCN	Subset A	DFCN	All	Subset A	0.97176
FCN	Subset A	DFCN	Subset A	Subset A	0.54821
FCN	Subset A	FCN	All	Subset A	0.33117
**FCN**	Subset A	RF	All	Subset A	**0.00339**
**FCN**	Subset A	RF	Subset A	Subset A	**0.01502**
FCN	Subset A	SDAE	All	Subset A	0.07703
FCN	Subset A	SDAE	Subset A	Subset A	0.39365
RF	All	DAE	All	Subset A	0.56853
RF	All	DAE	Subset A	Subset A	0.35180
RF	All	**DFCN**	All	Subset A	**0.00318**
RF	All	DFCN	Subset A	Subset A	0.08873
RF	All	**FCN**	All	Subset A	**0.02002**
RF	All	**FCN**	Subset A	Subset A	**0.00339**
RF	All	RF	Subset A	Subset A	0.05234
RF	All	SDAE	All	Subset A	0.14601
RF	All	**SDAE**	Subset A	Subset A	**0.01799**
RF	Subset A	DAE	All	Subset A	0.90902
RF	Subset A	DAE	Subset A	Subset A	0.79816
RF	Subset A	**DFCN**	All	Subset A	**0.01887**
RF	Subset A	DFCN	Subset A	Subset A	0.27054
RF	Subset A	FCN	All	Subset A	0.09254
RF	Subset A	**FCN**	Subset A	Subset A	**0.01502**
RF	Subset A	RF	All	Subset A	0.05234
RF	Subset A	SDAE	All	Subset A	0.47878
RF	Subset A	SDAE	Subset A	Subset A	0.07467
SDAE	All	DAE	All	Subset A	0.35907
SDAE	All	DAE	Subset A	Subset A	0.71616
SDAE	All	**DFCN**	All	Subset A	**0.02356**
SDAE	All	DFCN	Subset A	Subset A	0.50519
SDAE	All	FCN	All	Subset A	0.11815
SDAE	All	FCN	Subset A	Subset A	0.07703
SDAE	All	RF	All	Subset A	0.14601
SDAE	All	RF	Subset A	Subset A	0.47878
SDAE	All	SDAE	Subset A	Subset A	0.24057
**SDAE**	Subset A	DAE	All	Subset A	**0.02188**
SDAE	Subset A	DAE	Subset A	Subset A	0.17905
SDAE	Subset A	DFCN	All	Subset A	0.59272
SDAE	Subset A	DFCN	Subset A	Subset A	0.78887
SDAE	Subset A	FCN	All	Subset A	0.78720
SDAE	Subset A	FCN	Subset A	Subset A	0.39365
**SDAE**	Subset A	RF	All	Subset A	**0.01799**
SDAE	Subset A	RF	Subset A	Subset A	0.07467
SDAE	Subset A	SDAE	All	Subset A	0.24057

Table 8. p values from comparing AUC results (DeLong's test) of experiment Clinically relevant subsets A and B on **Subset B** labels of the test data. Models with statistical significance and significant p-values are presented in bold.

Model 1	Model 1 Training	Model 2	Model 2 Training	Test Dataset	p-value
DAE	All	DAE	Subset B	Subset B	0.98772
DAE	All	**DFCN**	All	Subset B	**0.00876**
DAE	All	DFCN	Subset B	Subset B	0.11224
DAE	All	FCN	All	Subset B	0.39230
DAE	All	FCN	Subset B	Subset B	0.15350
DAE	All	RF	All	Subset B	0.22107
DAE	All	RF	Subset B	Subset B	0.83053
DAE	All	SDAE	All	Subset B	0.80068
DAE	All	SDAE	Subset B	Subset B	0.12611
DAE	Subset B	DAE	All	Subset B	0.98772
DAE	Subset B	**DFCN**	All	Subset B	**0.00521**
DAE	Subset B	**DFCN**	Subset B	Subset B	**0.04597**
DAE	Subset B	FCN	All	Subset B	0.21183
DAE	Subset B	**FCN**	Subset B	Subset B	**0.04032**
DAE	Subset B	RF	All	Subset B	0.13714
DAE	Subset B	RF	Subset B	Subset B	0.77915
DAE	Subset B	SDAE	All	Subset B	0.65732
DAE	Subset B	**SDAE**	Subset B	Subset B	**0.01101**
**DFCN**	All	DAE	All	Subset B	**0.00876**
**DFCN**	All	DAE	Subset B	Subset B	**0.00521**
**DFCN**	All	DFCN	Subset B	Subset B	**0.00501**
**DFCN**	All	FCN	All	Subset B	**0.00108**
**DFCN**	All	FCN	Subset B	Subset B	**0.00801**
**DFCN**	All	RF	All	Subset B	< **0.000**
**DFCN**	All	RF	Subset B	Subset B	**0.00114**
**DFCN**	All	SDAE	All	Subset B	**0.00358**
DFCN	All	SDAE	Subset B	Subset B	0.12674
DFCN	Subset B	DAE	All	Subset B	0.11224
**DFCN**	Subset B	DAE	Subset B	Subset B	**0.04597**
DFCN	Subset B	**DFCN**	All	Subset B	**0.00501**
DFCN	Subset B	FCN	All	Subset B	0.17454
DFCN	Subset B	FCN	Subset B	Subset B	0.56112
**DFCN**	Subset B	RF	All	Subset B	**0.00019**
**DFCN**	Subset B	RF	Subset B	Subset B	**0.03065**
**DFCN**	Subset B	SDAE	All	Subset B	**0.04803**
DFCN	Subset B	SDAE	Subset B	Subset B	0.90291
FCN	All	DAE	All	Subset B	0.39230
FCN	All	DAE	Subset B	Subset B	0.21183
FCN	All	**DFCN**	All	Subset B	**0.00108**
FCN	All	DFCN	Subset B	Subset B	0.17454
FCN	All	FCN	Subset B	Subset B	0.19996
**FCN**	All	RF	All	Subset B	**0.00109**
FCN	All	RF	Subset B	Subset B	0.11613
FCN	All	SDAE	All	Subset B	0.09070
FCN	All	SDAE	Subset B	Subset B	0.05721
FCN	Subset B	DAE	All	Subset B	0.15350
**FCN**	Subset B	DAE	Subset B	Subset B	**0.04032**
FCN	Subset B	**DFCN**	All	Subset B	**0.00801**
FCN	Subset B	DFCN	Subset B	Subset B	0.56112
FCN	Subset B	FCN	All	Subset B	0.19996
**FCN**	Subset B	RF	All	Subset B	**0.00032**
**FCN**	Subset B	RF	Subset B	Subset B	**0.04280**
**FCN**	Subset B	SDAE	All	Subset B	**0.04172**
FCN	Subset B	SDAE	Subset B	Subset B	0.62502
RF	All	DAE	All	Subset B	0.22107
RF	All	DAE	Subset B	Subset B	0.13714
RF	All	**DFCN**	All	Subset B	< **0.000**
RF	All	**DFCN**	Subset B	Subset B	**0.00019**
RF	All	**FCN**	All	Subset B	**0.00109**
RF	All	**FCN**	Subset B	Subset B	**0.00032**
RF	All	**RF**	Subset B	Subset B	**0.01913**
RF	All	SDAE	All	Subset B	0.27330
RF	All	**SDAE**	Subset B	Subset B	**0.00021**
RF	Subset B	DAE	All	Subset B	0.83053
RF	Subset B	DAE	Subset B	Subset B	0.77915
RF	Subset B	**DFCN**	All	Subset B	**0.00114**
RF	Subset B	**DFCN**	Subset B	Subset B	**0.03065**
RF	Subset B	FCN	All	Subset B	0.11613
RF	Subset B	**FCN**	Subset B	Subset B	**0.04280**
**RF**	Subset B	RF	All	Subset B	**0.01913**
RF	Subset B	SDAE	All	Subset B	0.95579
RF	Subset B	**SDAE**	Subset B	Subset B	**0.02479**
SDAE	All	DAE	All	Subset B	0.80068
SDAE	All	DAE	Subset B	Subset B	0.65732
SDAE	All	**DFCN**	All	Subset B	**0.00358**
SDAE	All	**DFCN**	Subset B	Subset B	**0.04803**
SDAE	All	FCN	All	Subset B	0.09070
SDAE	All	**FCN**	Subset B	Subset B	**0.04172**
SDAE	All	RF	All	Subset B	0.27330
SDAE	All	RF	Subset B	Subset B	0.95579
SDAE	All	**SDAE**	Subset B	Subset B	**0.00596**
SDAE	Subset B	DAE	All	Subset B	0.12611
**SDAE**	Subset B	DAE	Subset B	Subset B	**0.01101**
SDAE	Subset B	DFCN	All	Subset B	0.12674
SDAE	Subset B	DFCN	Subset B	Subset B	0.90291
SDAE	Subset B	FCN	All	Subset B	0.05721
SDAE	Subset B	FCN	Subset B	Subset B	0.62502
**SDAE**	Subset B	RF	All	Subset B	**0.00021**
**SDAE**	Subset B	RF	Subset B	Subset B	**0.02479**
**SDAE**	Subset B	SDAE	All	Subset B	**0.00596**

References

1. Smith LN. Cyclical Learning Rates for Training Neural Networks. arXiv:150601186 [cs]. 2017;.

2. Szegedy C, Vanhoucke V, Ioffe S, Shlens J, Wojna Z. Rethinking the Inception Architecture for Computer Vision. arXiv:151200567 [cs]. 2015;.

3. Pedregosa F, Varoquaux G, Gramfort A, Michel V, Thirion B, Grisel O, et al. Scikit-learn: Machine Learning in Python. Journal of Machine Learning Research. 2011;12(85):2825–2830.
