# Supplementary material for: Deep learning with robustness to missing data: A novel approach to the detection of COVID-19
Source: PLoS One. 2021 Jul 30;16(7):e0255301. doi: 10.1371/journal.pone.0255301 (PMC8323880; doi:10.1371/journal.pone.0255301)
Supplement: Attachment — Submitted filename: Response to reviewer comments Predicting COVID-19 DFCN.pdf [file pone.0255301.s002.pdf]

Dear Editor,

Thank you for taking the time to manage the review process of our submission. In this letter, I'm sharing the changes that we've made to address all of the issues raised in the review process. Here I provide each comment followed by our response, highlighted in gray. When applicable, we provide the relevant changes in the manuscript. In this letter, we use italic font to indicate the parts from the manuscript where bold indicates the change. In the manuscript, bold font indicates changed text.

Kind regards,

Erdi Çallı

1. Please ensure that your manuscript meets PLOS ONE's style requirements, including those for file naming. The PLOS ONE style templates can be found at

https://journals.plos.org/plosone/s/file?id=wjVg/PLOSOne_formatting_sample_main_body.pdf
and

https://journals.plos.org/plosone/s/file?id=ba62/PLOSOne_formatting_sample_title_authors_affiliations.pdf

We use the latex template provided in https://journals.plos.org/plosone/s/latex. We believe that this adheres to the style guideline provided above.

2. Thank you for providing the date(s) when patient medical information was initially recorded. Please also include the date(s) on which your research team accessed the databases/records to obtain the retrospective data used in your study.

We have addressed this issue by adding this sentence in the Data subsection.

Data was shared with our institution on 28 May 2020.

3. We note that you have stated that you will provide repository information for your data at acceptance. Should your manuscript be accepted for publication, we will hold it until you provide the relevant accession numbers or DOIs necessary to access your data. If you wish to make changes to your Data Availability statement, please describe these changes in your cover letter and we will update your Data Availability statement to reflect the information you provide.

The data has been made available under the DOI 10.5281/zenodo.4461478.

4. Thank you for stating the following in the Acknowledgments Section of your manuscript:

'Funding for this study was partially provided by the Botnar Research Centre for Child Health.'

We note that you have provided funding information that is not currently declared in your Funding Statement. However, funding information should not appear in the Acknowledgments section or other areas of your manuscript. We will only publish funding information present in the Funding Statement section of the online submission form.

a. Please remove any funding-related text from the manuscript and let us know how you would like to update your Funding Statement. Currently, your Funding Statement reads as follows:

'The author(s) received no specific funding for this work.'

b. Please include your amended statements within your cover letter; we will change the online submission form on your behalf.

We have removed the acknowledgements section from the study. Please update the online submission to include this statement;

Funding for this study was partially provided by the Botnar Research Centre for Child Health.

5. Please ensure that you refer to Figure 7 in your text as, if accepted, production will need this reference to link the reader to the figure.

We have moved the relevant figure to a new supporting document file S1 Fig.pdf.

6. We note you have included a table to which you do not refer in the text of your manuscript. Please ensure that you refer to Table 4 in your text; if accepted, production will need this reference to link the reader to the Table.

Added the relevant mention in Supporting Information, S1 Table, Data Cleanup.
\paragraph{S1 Table.}{\bf Data Cleanup} \label{tab:appendix_cleanup} Data cleanup protocol thresholds and replacement values* ***are provided in Table~\ref{tab:data_cleanup_protocol}.***

7. Please include captions for your Supporting Information files at the end of your manuscript, and update any in-text citations to match accordingly. Please see our Supporting Information guidelines for more information: http://journals.plos.org/plosone/s/supporting-information

At the end of our document, we provide the section Supporting Information. Within that section, we provide all supporting information captions and refer to these captions in the text accordingly.

Reviewer #1: I have a few observations regarding this submission before accept.

Dear Dr. Islam, thanks a lot for taking the time to review our study. We tried to address all your observations to our best ability. We hope that these answers will satisfy you.

Comment #1: How does the proposed system handle missing data? A mathematical proof is needed.

The details of how the missing data is handled by the proposed system are given under the details of subsection **Denoising Fully Connected Network** (starting line 130)**.** In short, the last sentence of the first paragraph (**The model is trained with randomly masked inputs which, combined with this unique regularization method, instills a robustness to missing input data.)** explains how the system learns to deal with missing data. The details of the fundamental elements and necessary citations are provided in the following subsections just below this paragraph. We think that a mathematical proof showing why our proposed method works requires us to first prove why any given deep learning model works and this is neither possible nor feasible for the purposes of this study.

Comment #2: A proof to show the robustness of the architecture is highly needed.

We believe that the proof that shows the robustness of this architecture is already provided in our manuscript. In our **Results** section, the **Ablation study on independent test dataset** subsection details how this our proposed model compares to other models under various conditions. The statistical significance comparisons we show in **Figure 6** demonstrate that our proposed model outperforms every other model under all possible ranges of missing inputs. We believe that this comparison is the greatest challenge that we can provide to prove robustness of the model.

Comment #3: COVID-19 researches from different directions must be cited to properly present the background of the study.

Shah Muhammad Azmat Ullah, Md. Milon Islam, Saifuddin Mahmud, Sheikh Nooruddin, S. M. Taslim Uddin Raju and Md. Rezwanul Haque, “Scalable Telehealth Services to Combat Novel Coronavirus (COVID-19) Pandemic” SN Computer Science, Springer, vol. 2, no.1, pp. 18, 2020.

Md. Milon Islam, Saifuddin Mahmud, L. J. Muhammad, Md. Rabiul Islam, Sheikh Nooruddin and Safial Islam Ayon, “Wearable Technology to Assist the Patients Infected with Novel Coronavirus (COVID-19)," SN Computer Science, Springer, vol. 1, no. 6, pp. 320, Sep. 2020.

Md. Milon Islam, Shah Muhammad Azmat Ullah, Saifuddin Mahmud and S. M. Taslim Uddin Raju "Breathing Aid Devices to Support Novel Coronavirus (COVID-19) Infected Patients," SN Computer Science, Springer, vol. 1, no. 5, pp. 274, Aug. 2020.

Mohammad Marufur Rahman, Md. Motaleb Hossen Manik, Md. Milon Islam, Saifuddin Mahmud and Jong-Hoon Kim," An Automated System to Limit COVID-19 Using Facial Mask Detection in Smart City Network IEEE International IOT, Electronics and Mechatronics Conference (IEMTRONICS), IEEE, Vancouver, BC, Canada, pp. 1-5, 9-12 Sep., 2020.

We believe that these studies diverge too much from our work. There are more than 3000 studies out there that cover various aspects of COVID-19 and how it affects our lives. Our study is not intended to provide an overall view for all these studies.

Comment #4: Novelty is confusing. A highlight is required. The main contributions of the manuscript are not clear. The main contributions of the article must be very clear and would be better if summarize them into 3-4 points at the end of the introduction.

The final paragraph of the introduction has been updated to fully summarize the contributions of this work. It now reads as follows: *"To our knowledge, this is the first time the DFCN architecture has been proposed and evaluated. The useful properties of the architecture are explained through an ablation study and its novel benefits in terms of classification performance and robustness to missing data are demonstrated in this work. It is additionally applied to a globally important problem - diagnosis of COVID-19 - where issues of missing input data may be expected." (Please note that a further paragraph was added to address comment #11, so the text above is now in the second-last paragraph of the Introduction)*

Comment #5: The following references must be cited in Introduction section to describe the deep learning based systems for COVID-19 diagnosis.

M. M. Islam, F. Karray, R. Alhajj and J. Zeng, "A Review on Deep Learning Techniques for the Diagnosis of Novel Coronavirus (COVID-19)," in IEEE Access, doi: 10.1109/ACCESS.2021.3058537.

Amanullah Asraf, Md. Zabirul Islam, Md. Rezwanul Haque and Md. Milon Islam," Deep Learning Applications to Combat Novel Coronavirus (COVID-19) Pandemic," SN Computer Science, Springer, vol. 1, no. 6, pp. 363, Nov. 2020.

Muhammad Lawan Jibril, Md. Milon Islam, Usman Sani Sharif and Safial Islam Ayon, "Predictive Data Mining Models for Novel Coronavirus (COVID-19) Infected Patients Recovery," SN Computer Science, Springer, vol. 1, no. 4, pp. 206, Jun. 2020.

Reference to 10.1109/ACCESS.2021.3058537 is added in the Introduction section. The other two are not cited because they are not COVID-19 diagnosis related.

An overview of various methods to predict COVID-19 diagnosis is provided by \cite{Isla21}.

Comment #6: Methodology is not clear. Provide an algorithm and flowchart of the whole work. The authors need to add a new figure to show the main structure of the proposed system. This will help the reader to get a better understanding of what is going on in the proposed system.

The overall architecture of the various models is provided in Figure 2. In Figure 1, we explain how the reconstruction regularization is obtained. In Figures 3 and 4, we provide the key architectures used in this study. We believe that these provide the main structure of the proposed system.

Comment #7: Comparison with the following works are highly required.

Md. Zabirul Islam, Md. Milon Islam and Amanullah Asraf, "A Combined Deep CNN-LSTM Network for the Detection of Novel Coronavirus (COVID-19) Using X-ray Images," Informatics in Medicine Unlocked, Elsevier, vol. 20, pp. 100412, Aug. 2020.

Prottoy Saha, Muhammad Sheikh Sadi and Md. Milon Islam," EMCNet: Automated COVID-19 Diagnosis from X-ray Images using Convolutional Neural Network and Ensemble of Machine Learning Classifiers," Informatics in Medicine Unlocked, Elsevier, vol. 22, pp. 100505, Jan. 2021.

M. M. Islam, M. Z. Islam, A. Asraf, and W. Ding, “Diagnosis of COVID-19 from X-rays Using Combined CNN-RNN Architecture with Transfer Learning,” Aug. 2020. [Online]. Available: https://www.medrxiv.org/content/10.1101/2020.08.24.20181339v1.

Our proposed system uses Laboratory values next to Chest X-rays to be able to provide an RT-PCR diagnosis. We make comparisons with studies that use the same dataset [5] and a comparable method [11]. These studies use the RT-PCR test results as the gold standard reference. We believe that the studies mentioned by the reviewer can not be reasonably compared to our study because they only use chest X-ray readings as a reference.

Comment #8: Abstract is unnecessarily wordy. Make it brief and concise. Also, Conclusion should clearly state the outcome. Some of the obtained results need to be highlighted in the conclusion section.

To address this issue we have shortened the Abstract. Now it reads as follows

In the context of the current global pandemic and the limitations of the RT-PCR test, we propose a novel deep learning architecture, DFCN (Denoising Fully Connected Network). Since medical facilities around the world differ enormously in what laboratory tests or chest imaging may be available, DFCN is designed to be robust to missing input data. An ablation study extensively evaluates the performance benefits of the DFCN as well as its robustness to missing inputs.

Data from 1088 patients with confirmed RT-PCR results are obtained from two independent medical facilities. The data includes results from 27 laboratory tests and a chest x-ray scored by a deep learning model. Training and test datasets are taken from different medical facilities. Data is made publicly available.

The performance of DFCN in predicting the RT-PCR result is compared with 3 related architectures as well as a Random Forest baseline. All models are trained with varying levels of masked input data to encourage robustness to missing inputs. Missing data is simulated at test time by masking inputs randomly.

DFCN outperforms all other models with statistical significance using random subsets of input data with 2-27 available inputs.

When all 28 inputs are available DFCN obtains an AUC of 0.924, higher than any other model. Furthermore, with clinically meaningful subsets of parameters consisting of just 6 and 7 inputs respectively, DFCN achieves higher AUCs than any other model, with values of 0.909 and 0.919

Comment #9: There are lots of typos. English needs to revise again with a professional editing service. Also, the figures are not clear in some cases.

Our text has been co-authored and proof-read by native English speakers. On top of that, our non-native speakers use automated tools to check for typos in the manuscript. We notice that the town name **‘s Hertogenbosch** may be misunderstood as a typo, however it is indeed a correctly written town name in the Netherlands (for confirmation, please see: https://en.wikipedia.org/wiki/%27s-Hertogenbosch). Based on your comment we made another check using automated tools as well as our native speakers, however, we did not find any typos in our manuscript. If there are typos that were missed by this process, please let us know and we will be happy to fix them.

Comment #10: Mention the limitations and future works of the developed system elaborately.

We provided the limitations and future work in the Discussions section, between lines 400 and 413. The paragraph that starts from line 400 explains the limitations of our study. The following paragraph that starts from line 409 explains the future work on this topic. We feel that this text includes sufficient information and detail on these matters, but we are happy to elaborate if any detail has been overlooked here.

Comment #11: Please, add a small paragraph to describe the main structure of the manuscript at the end of the introduction.

We have addressed this issue by adding a paragraph at the end of introduction. It reads as follows

The remainder of the manuscript is structured as follows: The Data and Methods section defines the dataset, the details of DFCN, and the rest of the models in the ablation study. The Experiments section describes all experiments that were performed, and the results of these are presented in the subsequent Results section. The findings of these experiments are interpreted and discussed further in the Discussion section, which concludes the manuscript.

Comment #12: All the figures presented in the experimental results section are not discussed by the authors. This is a critical issue in this section.

In the results section, we provide two figures, Figure 5 and Figure 6. Figure 5 is discussed and explained under the Results section, lines 303, 333. Figure 6 shows the statistical significance of every point in Figure 5 and the results of Figure 6 are explained in the paragraph starting from line 306. Hence the paragraph that explains Figure 6 is also an explanation for Figure 5.

These results are further explained in detail under the Discussions section. First 6 paragraphs of the Discussion section form an extensive discussion of these results.

Comment #13: 10-fold cross-validation is adopted, right? Authors should state this in the main text explicitly.

10-fold cross validation is not adopted. In this study, we trained a model using data from one hospital and validated this model using completely independent data from another hospital. This is explained in the text in lines 96 to 98.

Comment # 14: Experiments with benchmark dataset (J. P. Cohen, P. Morrison, and L. Dao, “COVID-19 Image Data Collection,” 2020, arXiv: 2003.1159. [Online]. Available: https://arxiv.org/abs/2003.11597) are highly needed to proof the universal use cases.

The lack of laboratory parameters for the provided dataset makes it impossible for us to experiment with our method on this data.

Reviewer #2: the Subject was interesting and I prefer to publish it but there is a minor Revision that I am not convinced about the performance of the models. the authors don't mention anything about Overfitting the model. the ROC chart doesn't show in the manuscript and it's an important problem. In table 3 (Comparison of models on the test dataset) it's better to show the models chart to compare their AUC and another metric. it's conventional to show details of confusion matrix metrics including(Sensitivity and Specificity).

Dear Dr. Ghaderzadeh, thanks a lot for taking the time to review our study. We tried to address all your observations to our best ability. We hope that these answers will satisfy you.

The authors don't mention anything about Overfitting the model.

Your point on this topic is very valid. We believe that by testing our models on a dataset from a different institution (than the training dataset), we have prevented the implications of overfitting on our models. We have addressed this issue by elaborating on it in the Data and Methods section.

Another place where overfitting had an implication is, we train our Chest X-ray model with one dataset, and we use the predictions extracted from this model and its training dataset as a parameter for the laboratory values models. We addressed this issue by explicitly stating the choices that we have done to prevent overfitting in Experiments and Supplementary Materials sections. The changes read as follows

Data section
By testing on data from a different institution we demonstrate that our method is generalizable and not overfitted to characteristics of the data from a single institute.

Experiments section

To prevent overfitting, *we re-initialize and fine-tune only the last layer of this model on the training dataset from BHH to predict the RT-PCR test results and apply softmax activations to obtain likelihood of positive and negative outcomes.*

Supplementary Information
When fine-tuning the last layer on RT-PCR test results, we use a heavy label smoothing regularization \cite{Szeg15} of 0.2 ***to prevent overfitting.***

Before we feed those images to the convolutional neural network, we apply data augmentation to prevent overfitting. *We augment the images by cropping a width and height in the range of (409, 512] randomly. We resize the resulting image to 448 by 448. We randomly scale (by $[0.75, 1.25]$) and shift (± 64) the pixel values and clip the values above 255. We randomly flip the image from left to right.*

The ROC chart doesn't show in the manuscript and it's an important problem. In table 3 (Comparison of models on the test dataset) it's better to show the models chart to compare their AUC and another metric. it's conventional to show details of confusion matrix metrics including(Sensitivity and Specificity).

We have addressed this issue by including the ROC curves of the DFCN in the results section.

The ROC curves of the highest performing model from each section of Table 3 are provided in Figure 7. The p values for all model comparisons in these experiments are presented in Supporting Information S3 Table.

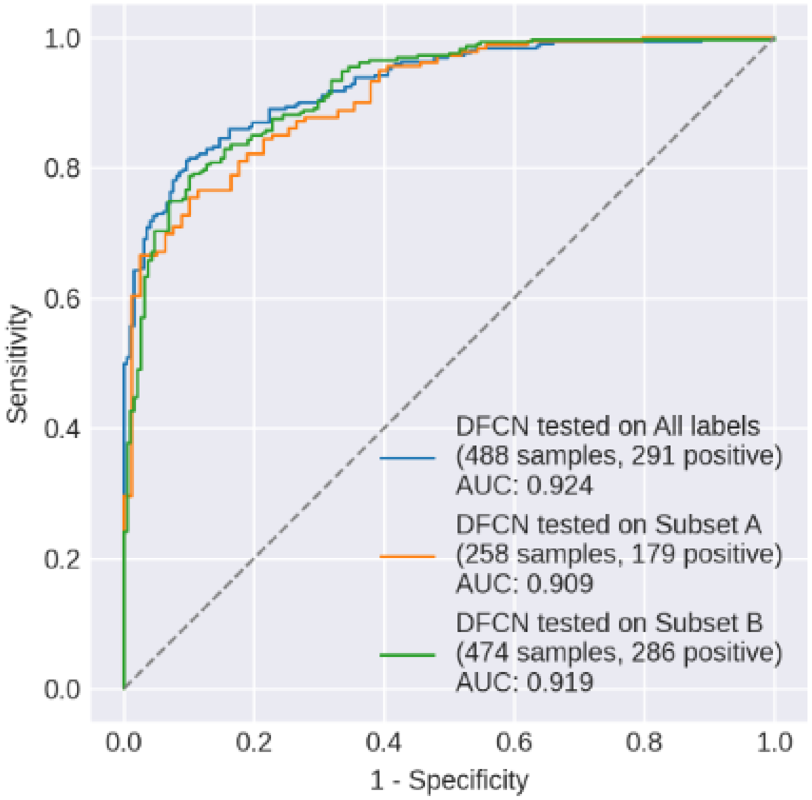

Fig 7. Comparison of the ROC curves of DFCN for all available inputs, for the Subset A and the Subset B.
